# Supplementary material for: Resolving the full spectrum of human genome variation using Linked-Reads
Source: Genome Res. 2019 Apr;29(4):635–45. doi: 10.1101/gr.234443.118 (PMC6442396; doi:10.1101/gr.234443.118)
Supplement: Supplemental Material [file supp_gr.234443.118_Supplemental_longranger-2.2.zip › longranger-2.2.tar.bz2!/lr2.2-public/lib/stan/cmdstan/stan/doc/api/html/structstan_1_1io_1_1stan__csv__timing-members.html]

Stan: Member List


|  |  |
| --- | --- |
|  | Stan  2.5.0  probability, sampling & optimization |


- Main Page
- Related Pages
- Namespaces
- Classes
- Files

- Class List
- Class Index
- Class Hierarchy
- Class Members

All Classes Namespaces Files Functions Variables Typedefs Enumerations Enumerator Friends Macros Pages

- stan
- io
- stan\_csv\_timing

stan::io::stan\_csv\_timing Member List

This is the complete list of members for stan::io::stan\_csv\_timing, including all inherited members.

|  |  |  |
| --- | --- | --- |
| sampling | stan::io::stan\_csv\_timing |  |
| stan\_csv\_timing() | stan::io::stan\_csv\_timing | inline |
| warmup | stan::io::stan\_csv\_timing |  |


---

[ Stan Home Page ]

*© 2011–2014,
Stan Development Team.*
